# A novel homozygous CA5A gene deletion in carbonic anhydrase VA deficiency presenting as developmental delay without metabolic crisis

**DOI:** 10.1016/j.ymgmr.2026.101294

**Published:** 2026-02-17

**Authors:** Maryam F. Bin Hadyan, Mohammed A. Saleh, Saad Aldalaqan, Aziza M. Mushiba, Ali M. Alasmari, Eissa A. Faqeih, Abdul A. Peer-Zada

**Affiliations:** a Department of Clinical and Metabolic Genetics, Pediatric Hospital, King Fahad Medical City, Riyadh, Saudi Arabia; b Department of Pathology and Clinical Laboratory Medicine Molecular Pathology Section, King Fahad Medical City, Riyadh, Saudi Arabia

**Keywords:** Carbonic anhydrase VA deficiency, CA5A gene, Developmental delay, Metabolic disorders, Autosomal recessive disease, Whole genome sequencing, Whole exome sequencing

## Abstract

**Background:**

Carbonic anhydrase VA deficiency is a rare autosomal recessive disorder caused by biallelic mutations in the CA5A gene. Patients present with acute metabolic decompensation including hyperammonemia in infancy albeit a good outcome.

**Objective:**

We report three children from the same Saudi tribe with a novel homozygous deletion in CA5A gene, manifesting predominantly as developmental delay without hyperammonemia and major metabolic crises.

**Methods:**

Diagnostic work-up included clinical, biochemical, neuroimaging, and genetic analyses through WES and WGS with family segregation analysis.

**Results:**

The first patient, a 3-year-old girl, presented with global developmental delay, corpus callosum thinning, and mild periventricular leukomalacia on brain MRI. The second patient, a 7-year-old girl born to consanguineous parents, had delayed motor and language milestones with persistent speech delay, microcephaly, and mild to moderate intellectual disability, but normal metabolic and neuroimaging findings. Her younger sister, aged 4 years, showed mild speech delay without additional clinical abnormalities with biochemical investigations in both siblings unremarkable. None presented with classic neonatal hyperammonemia. A pathogenic homozygous loss of 16.5 kb (exons 3–7) in CA5A gene (chr16:87921735–87,938,510 NM_001739.2) was identified in all the three children with the parents and healthy siblings carrying the variant in heterozygous state.

**Conclusion:**

CA-VA deficiency may present with non-specific neurodevelopmental delay without metabolic decompensation. Genetic analysis remains the cornerstone for identifying atypical cases with novel mutations in a rare disease and recognition of this atypical presentation is essential for awareness of the disease.

## 1 Introduction

Carbonic anhydrase VA (CA-VA) deficiency is an autosomal recessive metabolic disorder caused by pathogenic variants in the CA5A gene (OMIM#114761). The CA5A gene encodes an intramitochondrial carbonic anhydrase, which belongs to a large family of zinc metalloenzymes that catalyze the reversible hydration of carbon dioxide pivotal for providing bicarbonate (HCO3-) for multiple mitochondrial enzymes [1]. They participate in a variety of biological processes, including respiration, calcification, acid-base balance, bone resorption, and the formation of aqueous humor, cerebrospinal fluid, saliva, and gastric acid [2]. They show extensive diversity in tissue distribution and in their subcellular localization in the mitochondria and expressed primarily in the liver [3]. Clinically, CA-VA deficiency is characterized by acute metabolic decompensation in infancy or early childhood, with features such as metabolic acidosis, hyperammonemia, hypoglycemia, and lactic acidosis [4]. Other abnormalities include hypernatremia, increased serum lactate and alanine, ketosis, metabolic distress, respiratory alkalosis, seizures, encephalopathy and impaired provision of bicarbonate to essential mitochondrial enzymes. The clinical course is usually benign except acute crisis in neonatal period [[5], [6], [7], [8], [9], [10], [11], [12], [13], [14], [15], [16]].

We report three children from the same Saudi tribe carrying a novel homozygous pathogenic CA5A exon 3–7 deletion manifesting predominantly as developmental delay without hyperammonemia and major metabolic crises. These are the first reported cases of CA-VA deficiency in Saudi Arabia with a novel variant in CA5A gene.

## 2 Methods

All patients were managed at a tertiary care pediatric hospital within King Fahad Medical City. Clinical, biochemical, and neuroimaging data were collected from electronic medical records. Genetic testing was performed using whole exome (WES) and genome sequencing (WGS), with family segregation studies in affected families. Informed consent for genetic analysis and publication was obtained from parents. The study was approved by the Institutional Ethics Committee.

## 3 Case presentations

### 3.1 Family 1 (case#1)

**Fig. 1:**
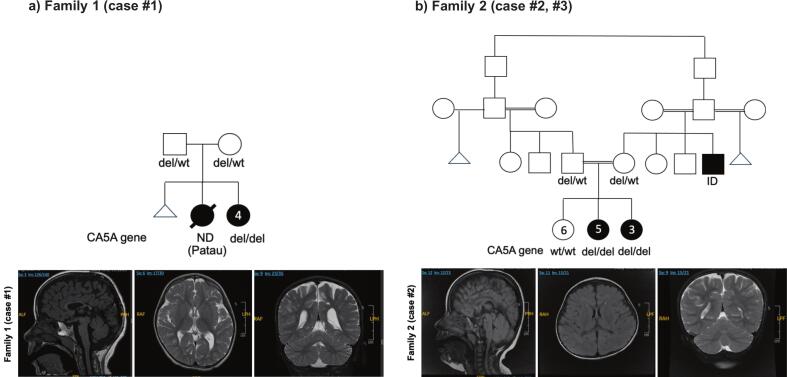
Pedigree and brain MRI of the index case in each family. A) and B), family 1 (case #1) and family 2 (case #2, #3) showing affected cases (solid boxes) and unaffected (open boxes) with CA5A gene variants (homozygous as del/del and heterozygous as del/wt; wild-type or normal). Ages of the cases are shown inside each circle or box; C) and D), MRI of the brain with Sagittal and axial reconstruction showing white matter disease with mild periventricular leukomalacia and thinning of the corpus callosum.

A 3-year-old female, born to a non-consanguineous couple from the same tribe (Fig. 1A pedigree) was referred for evaluation of global developmental delay. At the age of three years, she was able to sit and crawl but had not achieved independent walking, and she also had significant language delay. Her medical history was otherwise unremarkable, with no prior hospitalizations and no episodes suggestive of metabolic acidosis, hyperammonemia, or hypoglycemia. Growth parameters were within normal limits, and she had no dysmorphic features, although neurological examination revealed axial hypotonia. Biochemical investigations showed a normal plasma ammonia level of 41 μmol/L, a slightly elevated lactate of 3.73 mmol/L, and an unremarkable tandem mass spectrometry profile (Table 1). Brain MRI demonstrated moderately marked thinning of the corpus callosum along with mild periventricular leukomalacia (Fig. 1B). WES confirmed a pathogenic homozygous deletion involving exons 3–7 of the CA5A gene (chr16:87921735–87,938,510 NM_001739.2), consistent with autosomal recessive CA-VA deficiency.

**Table 1:** Biochemical findings observed in our patients in comparison with reported cases.

Analyte	Reference range	Family 1 (case #1)	Family 2 (case #2)	Family 2 (case #3)	Published literature
Ammonia	18–72 (μmol/l)	41	30	40	very high
Lactate	0.5–2.2 (mmol/L)	3.73	1.17	1.8	high
DBS acylcarnitine, amino acid (MS/MS)	-(μmol/L)	unremarkable	unremarkable	unremarkable	elevated
Organic acid urine (GCMS)	-(μmol/L)	unremarkable	unremarkable	unremarkable	elevated ketone bodies
Anion Gap	-(mmol/L)	24.5	13.7	11.1	acidosis/alkalosis
Bicarbonate (CO2), plasma	20–28 (mmol/L)	13.2	18.6	26.2	acidosis/alkalosis

### 3.2 Family 2 (case# 2 and #3)

The second patient was a female who first presented at three years of age and is currently seven years old. She was born to consanguineous parents, who were second cousins (Fig. 1C pedigree). The family reported delayed attainment of both motor and language milestones. Although she eventually caught up with her motor skills, she continued to have significant speech delay, accompanied by mild to moderate intellectual disability. She had no history of hospital admissions and no clinical features of metabolic acidosis, hyperammonemia, or hypoglycemia. On examination, her growth parameters were appropriate for age except for a head circumference below the third percentile, consistent with microcephaly. She was otherwise non-dysmorphic and had an unremarkable systemic examination. Neurometabolic investigations, including tandem mass spectrometry, urine organic acids, plasma amino acids, creatine kinase, liver and renal function tests, ammonia, and lactate, were all normal (Table 1). Brain MRI revealed minor abnormalities (Fig. 1D). WGS identified a pathogenic homozygous deletion involving exons 3–7 of the CA5A gene (chr16:87921735–87,938,510 NM_001739.2).

**Fig. 2:**
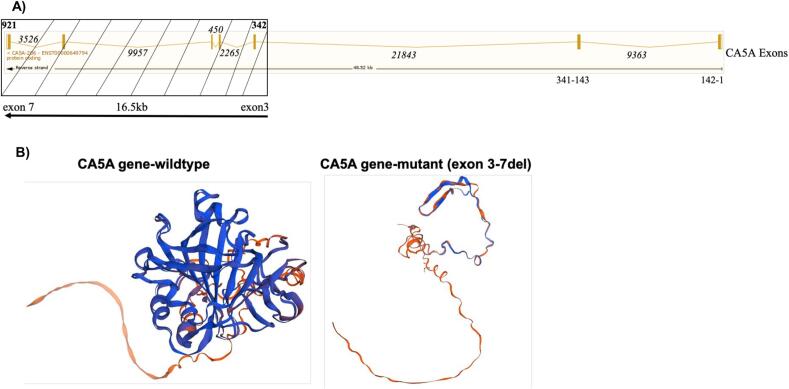
Exon map of CA5A gene and 3D protein structure comparison. A), detailed exon map (reverse strand) of the *CA5A* gene showing all the 7 exons and 16.5 kb exon 3–7 deletion (box with stripes). The numbers are nucleotide positions of exons and introns. B), 3D structures obtained for wild type CA5A gene and the mutant (exon 3–7 deletion) using the Swissmodel (swissmodel.expasy.org) prediction.

The third patient was the younger sister of Case 2, a 4-year-old female, who was evaluated for developmental concerns. She had mild developmental delay, predominantly affecting speech, but otherwise exhibited normal gross motor development. Like her sister, she had never been hospitalized and had no documented episodes of metabolic crises, including hypoglycemia or encephalopathy. On clinical assessment, she was non-dysmorphic, with normal growth and systemic examination. Biochemical investigations, including plasma ammonia, lactate, tandem mass spectrometry, and urine organic acids, were within normal limits. Genetic testing confirmed that she was homozygous for the same CA5A exon 3–7 deletion as her sister. Family segregation studies revealed that both parents were heterozygous carriers of the variant, while other healthy siblings were wild type and heterozygous carrier. The exon map of the CA5A gene and the deletion is shown (Fig. 2A). 3D protein structure prediction using the Swissmodel (swissmodel.expasy.org) showed profound differences in the structure between the wildtype and the mutant (exon 3–7 deletion) CA5A gene (Fig. 2B).

WGS data analysis also revealed two other likely pathogenic variants in SYT2 (c.797_801 + 1del; p.?) and SYNE4 (c.355C > T;p.R119W) genes: index (#2) had SYT2 (homozygous) and SYNE4 (heterozygous); affected sister (#3) had SYT2 (homozygous) and SYNE4 (homozygous); healthy sister, the mother and the father had SYT2 (heterozygous) and SYNE4 (heterozygous) gene variants. SYT2 causes autosomal dominant/autosomal recessive congenital myasthenic syndrome 7 A/7B (OMIM#600104), and SYNE4 causes autosomal recessive deafness (OMIM#615535). There is lack of genotype-phenotype correlation and therefore, these variants may be considered as secondary findings.

## 4 Discussion

CA-VA deficiency, an extremely rare inborn error of metabolism is characterized by acute metabolic crisis in the neonatal period and is a major cause of hyperammonemia in such patients, which in turn may lead to diagnostic ambiguity with other potential differential diagnosis such as transient hyperammonemia of the newborn, pyruvate carboxylase deficiency and multiple carboxylase deficiency. Till date about 46 cases of CA-VA deficiency have been reported in the literature from multiple ethnicities (Table 2) such as the Indian subcontinent (India, Pakistan, Sri Lanka & Bangladesh; 16/46), Oman/UAE (18/46), Turkish (1/46), Russian (3/46), Belgian-Scottish (4/46), Central America (1/42) and unreported (3/46) [1,4,[5], [6], [7], [8], [9], [10], [11], [12], [13], [14], [15], [16]]. We report for the first time, non-specific neurodevelopmental delay without hyperammonemia and metabolic crisis in Saudi patients with CA-VA deficiency confirmed by a novel bi-allelic mutations in CA5A gene. The classical neonatal phenotype described previously indicated severe hyperammonemic crises, encephalopathy, metabolic acidosis and respiratory alkalosis [1]. On the contrary, our patients showed persistent developmental delay with unremarkable biochemical profiles indicating no acidosis, alkalosis or ketosis, albeit with a homozygous deletion in the CA5A gene. A study by van Karnebeek et al. described 3 children from 2 unrelated families who presented with acute lethargy, tachypnea associated with severe hyperammonemia, lactic acidosis, respiratory alkalosis, hypoglycemia, and secondary carboxylase enzyme dysfunction [1]. None of these features were present in our patients. They further report episodic acute metabolic decompensation, mild axial hypotonia with below average motor coordination but with normal development. One of the patients in their study showed good developmental progress with mild learning difficulties, while his older brother who carried the same homozygous mutation, was unaffected at age 17 years. Our patients also showed mild axial hypotonia which resolved with time, reduced psychomotor development and learning difficulties.

**Table 2:** Notable CA5A gene variants reported in the literature from multiple ethnicities.

Reference	*Current (2025)*	*Ref* [10] *(2025)*	*Ref* [6] *(2025)*	*Ref* [16] *(2024)*	*Ref* [9] *(2024)*	*Ref* [8] *(2022)*	*Ref* [15] *(2022)*	*Ref* [14] *(2022)*	*Ref* [13] *(2021)*
Number of patients	3	1	1	18	1	1	1	1	1
Ethnicity	Saudi	Guatemalan	Indian	Omani	Indian	Russian	Unknown (UK)	Indian	Indian
CA5A Variant (NM_001739.2)	Exon 3–7 del	c.475 T > C; p.W159R	Exon 6 del	c.59G>; p.W20*	Exon 6 del c.721G > A; p.E241K:	c.555G > A; p.K185K	Exon 6 del	c.59G > A; p.W20*	c.123G > A; p.W41*; c.690C > T; p.W230W
Type of variant	Gross Deletion	Missense	Deletion	Nonsense	Missense, Deletion	Synonymous	Deletion	Non-sense	Nonsense, Synonymous
Zygosity	Homozygous	Homozygous	Homozygous	Homozygous	Compound heterozygous	Homozygous	Homozygous	Homozygous	Compound Heterozygous
Pathogenicity	Pathogenic	L.P	Pathogenic	Pathogenic	Pathogenic	L.P	Pathogenic	Pathogenic	Pathogenic, Likely Benign
ACMG Classification	PM2, PM3, PP3 PVS1, PS3	PM2, PP3, PP4	PM2, PM3, PP3, PP4	PVS1, PM2, PP4	PS3, PM2. PP3, PP4	PM2, PP3, PP5, PP4	PM2, PM3, PP3, PP4	PVS1, PM2, PP4	PVS1, PM2, PP4; PM2, BP6, BP7
Developmental delay	YES	NO	NO	YES	NO	NO	NO	YES	NO
Hyperammonemia	NO	YES	YES	YES, NO (*n* = 2)	YES	YES	YES	YES	YES
Hyperlactatemia	NO	YES	YES	YES	YES	YES	YES	YES	YES
Metabolic acidosis	NO	YES	YES	YES	YES	YES	YES	NO	YES
Ketosis	NO	YES	YES	YES	YES	YES	YES	NO	YES
HyperCKemia	NO	YES	NR	YES	NR	YES	NR	NR	NR
Hypoglycemia	NO	YES	YES	NO	NO	YES	NO	YES	NO


Reference	*Ref* [5] *(2020)*	*Ref* [7] *(2020)*	*Ref* [11] *(2020)*	*Ref* [12] *(2020)*	*Ref* [4] *(2016)*	*Ref* [1] *(2014)*
Number of patients	2	1	3	1	10	4
Ethnicity	Indian, Srilankan	Unknown (Germany)	Indian	Unknown (Turkey)	Turki, Indian, Russian, Pakistani, Belgian, Bangali	Belgian, Russian, Pakistani
CA5A Variant (NM_001739.2)	c.721G > A; p.E241K; Exon 6 del	c.721G > A; p.E241K	c.721G > A: c.788G > A and c.868C > T: Exon 1 del	c.721G > A; p.E241K	c.697 T > C; p.S233P: c.721G > A;p.E241K: Exon 6 del: c.555G > A-skipping of exon 4: c.123G > A; p.W41*: c.458_459 + 22del: c.555 + 4_555 + 183del	c.697 T > C: c.555G > A-skipping of exon 4: Exon 6 del: c.619_851del
Type of variant	Missense, Deletion	Missense	Deletion	Missense	Missense, Deletion	Missense, Deletion
Zygosity	Compound Heterozygous	Homozygous	Homozygous	Homozygous	Homozygous	Homozygous
Pathogenicity	Pathogenic	Pathogenic	Pathogenic	Pathogenic	Pathogenic	Pathogenic
ACMG Classification	PM2, PM3, PS3, PP3, PP4	PM2, PM3, PS3, PP3, PP4	PM2, PM3,PS3, PP3, PP4	PM2, PM3, PS3, PP3, PP4	PM2, PP3,PP5, PP4, PVS1	PM2, PP3, PP5, PP4, PVS1
Developmental delay	NO	NO	NO	NO	NO	NO
Hyperammonemia	YES	YES	YES	YES	YES	YES
Hyperlactatemia	YES	YES	YES	YES	YES	YES
Metabolic acidosis	YES	YES	YES	YES	YES	YES
Ketosis	YES	NR	NO	YES	YES	YES

Diez-Fernandez et al. (2016) reported 10 patients with homozygous mutations in CA5A gene, all with hyperammonemia, elevated lactate, and elevated ketone bodies in urine. The age of onset ranged from 2 days to 20 months. The authors concluded that CA-VA deficiency is a differential diagnosis of early onset and life-threatening metabolic crisis, with hyperammonemia, hyperlactatemia, and ketonuria as obligate signs [4]. Marwaha et al. report two cases with South Asian ancestry who presented with a metabolic decompensation characterized by hyperammonemia, lactic acidosis and ketonuria [5], and they suggest these as pathognomonic for CA-VA deficiency. Abdulwahab et al. reported a case of a female neonate of Indian origin presenting with hyperammonemia, severe lactic acidosis, hypoglycemia, and ketosis, and who received continuous renal replacement therapy [6]. Baertling F et al. reported fatal metabolic decompensation in carbonic anhydrase VA deficiency despite early treatment and normalization of ammonia and lactate levels during metabolic crisis [7]. Al-Thihli K et al. reported 18-Omani patients with a founder mutation, c.59G>A p.(Trp20*) in CA5A gene, sixteen of which presented with atypical clinical features including recurrent hyperammonemia, hyperCKemia, microcephaly, failure to thrive, developmental delay, and two patients were asymptomatic with normal ammonia levels [16]. Another case report showed hypoammonemia in CAVA-deficient neonate [17]. These studies indicate variable presentations in CA5A deficiency that may not necessarily involve typical hyperammonemia.

Brain MRI is one of the recommended investigations in CA-VA deficiency to define the extent of brain disease or edema. MRI in our patient revealed moderate thinning of the corpus callosum and periventricular leukomalacia. In the cases reported by Clara D. van Karnebeek et al. brain MRI and MRS on day 5 of life revealed a small periventricular petechial focus near the trigone of the right lateral ventricle, with a small lactate peak on spectroscopy in the male sibling and both MRI and EEG were unremarkable in the female sibling. Unremarkable brain MRI was also revealed by others, although cranial MRI revealed severe brain herniation [7].

WGS in our patients revealed a homozygous loss of 16.5 kb (exons 3–7) in CA5A gene (chr16:87921735–87,938,510 NM_001739.2) with the segregation analysis in the parents and healthy siblings revealing a carrier status in all of them except one healthy sibling with wild type CA5A gene. This confirmed the pathogenicity of the variant (ACMG criteria PM2, PM3, PVS1, PS3, PP3) and the diagnosis of CA-VA deficiency, which is caused by bi-allelic mutations in CA5A gene mapping to chromosome 16q24.3. Multiple mutations that include missense, splicing, and deletions have been reported in CA5A gene (Table 1) [1,4,[5], [6], [7], [8], [9], [10], [11], [12], [13], [14], [15], [16]]. Exon 3–7 deletion in our patients has not been reported before.

It is important to note here that quad WGS was performed in the consanguineous family due to the fact that targeted Sanger sequencing may pose a challenge to detect CA5A gene mutation in the heterozygous state. This is attributed to the presence of an unprocessed pseudogene CA5AP1 assigned to 16p12-p11.2 that has sequences homologous to exon 3–7 and introns 3–6 [3]. Thus, clinical laboratories, clinical geneticists and genetic counselors should be aware of this technical consideration when dealing with pseudogenes, and accordingly recommend appropriate follow-up genetic test or variant confirmation.

CA5A gene (Transcript: ENST00000649794.3 CA5A-206, chr16: 87,888,013-87,936,529 NM_001739.2) has 7 coding exons with 1113 bps transcript length and 305 amino acid residues. It comprises two domains: the alpha-CA domain, which spans from amino acid position 33 to 296; and the CA alpha-class, conserved site (within the aforementioned domain), which spans from amino acid position 141 to 157. Both domains are important for catalyzing the reversible hydration of carbon dioxide to bicarbonate. Exon 3–7 contains the metal-binding, active-site residues and the substrate-binding regions and its deletion is expected to present with severe phenotype and metabolic decompression. While one of our patients did present with white matter brain abnormalities, the others did not, and there was no major metabolic decompression. This could be attributed to compensatory mechanism through CA-VB or role of genetic modifiers and polygenic background as postulated by the study on Omani patients [16]. The pathomechanisms involved in CA-VA need further investigation.

In conclusion, CA-VA deficiency should be considered in Saudi children presenting with unexplained developmental delay, especially when accompanied by episodic vomiting or mild hyperammonemia. Routine metabolic screening and early use of genetic testing for identifying atypical cases with novel mutations can significantly improve outcomes. Furthermore, definitively attributing the developmental delay to CA5A deletion needs a larger number of cases and functional studies. Increased awareness and recognition of this clinical presentation is undoubtedly warranted.

## Author contribution

MH is a Fellow Geneticist who collected clinical data and wrote initial draft.

MS is a Clinical Geneticist involved in writing initial draft and clinical management of patient.

AM is a Clinical Geneticist involved in editing initial draft and clinical management of patient.

SD is a laboratory Clinical Scientist involved in data analyses, editing.

AA is a Clinical Geneticist involved in editing and clinical management of patient.

EF is a Clinical Geneticist involved in editing and clinical management of patient.

PZAA is a Laboratory Clinical Geneticist involved in conceptual design, writing of the manuscript.

## CRediT authorship contribution statement

**Maryam F. Bin Hadyan:** Writing – original draft, Methodology, Formal analysis, Data curation. **Mohammed A. Saleh:** Data curation. **Saad Aldalaqan:** Writing – review & editing. **Aziza M. Mushiba:** Visualization, Investigation. **Ali M. Alasmari:** Writing – review & editing, Investigation. **Eissa A. Faqeih:** Writing – review & editing, Validation, Supervision. **Abdul A. Peer-Zada:** Writing – review & editing, Writing – original draft, Supervision, Formal analysis, Conceptualization.

## Funding

None.

## Declaration of competing interest

The authors declare that there is no conflict of interest regarding the publication of this article.

## Data Availability

Data will be made available on request.
